# Reply to the letter from A. Zorzi and D. Corrado. Rhythm analysis with an AED in an unconscious athlete

**DOI:** 10.1007/s12471-018-1100-x

**Published:** 2018-03-08

**Authors:** N. M. Panhuyzen-Goedkoop, H. J. Wellens, J. J. Piek

**Affiliations:** 1 AMC Heart Center, Academic Medical Center, Amsterdam, The Netherlands; 2 Sports Medical Center Papendal Arnhem, Arnhem, The Netherlands; 3 Radboud University Medical Center, Nijmegen, The Netherlands; 4 The Cardiovascular Research Center, Maastricht, The Netherlands

We thank our colleagues Zorzi and Corrado for their comments on the problem of delayed on-site cardiopulmonary resuscitation and defibrillation with an AED when the athlete loses his/her consciousness during exercise [1, 2]. Those problems are well illustrated in their video from episodes of sudden cardiac arrest (SCA) in 22 athletes. They correctly stress the confusion created when agonal breathing and limb movements may mistakenly be interpreted that the person is still alive [1]. We agree with Zorzi and Corrado that the immediate use of an AED would allow recognition of a cardiac cause of unconsciousness, i. e. ventricular tachycardia or fibrillation or asystole [1]. Indeed, immediate cardiac massage and rhythm analysis with an AED should be performed as soon as possible in an unexpected unconscious athlete.
